# Validation of automatic wear-time detection algorithms in a free-living setting of wrist-worn and hip-worn ActiGraph GT3X+

**DOI:** 10.1186/s12889-019-6568-9

**Published:** 2019-02-28

**Authors:** Raphael Knaier, Christoph Höchsmann, Denis Infanger, Timo Hinrichs, Arno Schmidt-Trucksäss

**Affiliations:** Division Sports and Exercise Medicine, Department of Sport, Exercise and Health, University of Basel, Birsstrasse 320B, 4052 Basel, Switzerland

**Keywords:** Accelerometer, Physical activity, Free-living

## Abstract

**Background:**

Wrist-worn accelerometers are increasingly used in epidemiological studies to record physical activity. The accelerometer data are usually only analyzed if the convention for compliant wear time is met (i.e. ≥ 10 h per day) but the algorithms to detect wear time have been developed based on data from hip-worn devices only and have not been tested in a free-living setting. The aim of this study was to validate the automatic wear time detection algorithms of one of the most frequently used devices in a free-living setting.

**Methods:**

Sixty-eight adults wore one ActiGraph GT3X+ accelerometer on the wrist and one on the hip and additionally recorded wear times for each device separately in a diary. Monitoring phase was during three consecutive days in a free-living setting. Wear time was computed by the algorithms of Troiano and Choi and compared to the diary recordings.

**Results:**

Mean wear time was over 1420 min per day for both devices on all days. Lin’s concordance correlation coefficient for the wrist-worn wear time was 0.73 (0.60; 0.82) when comparing the diary with Troiano and 0.78 (0.67; 0.86) when comparing the diary with Choi. For hip-worn devices the respective values were 0.23 (0.13; 0.33) for Troiano and 0.92 (0.88; 0.95) for Choi. Mean and standard deviation values for absolute percentage errors for wrist-worn devices were − 1.3 ± 8.1% in Troiano and 0.9 ± 7.7% in Choi. The respective values for hip-worn devices were − 17.5 ± 10% in Troiano and − 0.8 ± 4.6% in Choi.

**Conclusions:**

Hip worn devices may be preferred due to their higher accuracy in physical activity measurement. Automatic wear-time detection can show high errors in individuals, but on a group level, type I, type II, and total errors are generally low when the Choi algorithm is used. In a real-life setting and participants with a high compliance, the algorithm by Choi is sufficient to distinguish wear time from non-wear time on a group level.

## Background

The measurement of physical activity as an important health parameter is a widely-used procedure in public health. In studies, objective methods such as accelerometers are often favored over subjective methods (i.e. questionnaires) to assess physical activity because self-reports of physical activity have been shown to overrate true values by up to 28% in males and 40% in females [1]. However, hip-worn accelerometers are not always worn reliably enough to accurately assess true levels of physical activity because of discomfort or simply because participants forget to wear them. This leads to false results or misinterpretation of the data. It has been shown that participants’ compliance expressed as a high wear-time in the long term, for example during intervention studies, is higher if the device is to be worn on the wrist instead of the hip due to the greater comfort and lower hindrance of the daily routine [2]. A recent study showed that 98.6% of participants wearing wrist-worn devices but only 91.0% of participants wearing hip-worn devices fulfil the criteria of 600 min wearing time per day [3].

Because they have a higher compliance, wrist-worn devices are increasingly used in research to assess physical activity in large-scale studies [4, 5]. Measurement of physical activity and energy expenditure by wrist-worn devices have been validated. However, as stated in the UK-Biobank study [4], a limitation of wrist-worn devices is that the optimal method to identify non-wear time remains elusive. A recent systematic review, which included all publications between January 2010 and December 2015 that are listed in PubMed and Web of Science and used the ActiGraph GT3X+ in their studies, analyzed the device with regard to data collection and data processing [6]. The authors provide some guidance on how activity data should be recorded and how activity should be categorized and analyzed. However, regarding non-wear time detection, the authors stated that *“There is a need to thoroughly test this criterion”* (Migueles et al., 2017, p. 1823). The matter of a missing validation concerns both wrist-worn and hip-worn devices and a valid wear-time recognition is crucial to distinguish non-wear time from sedentary behavior. Especially sedentary behavior needs to be detected correctly in epidemiological studies because it is an important health risk factor. In addition, participants’ wear-time is an important criterion to determine if data should be included in analyses or if data have to be excluded because of participants’ non-compliance. Conventionally, wear times ≥10 h per day are considered compliant wear [7]. According to a review [8], 51% of large-scale studies (participants: *n* > 400) used ActiGraph (Pensacola, United States) accelerometers which makes it one of the most-used tools to measure physical activity. In all ActiGraph devices, two different algorithms can be selected to identify wear and non-wear time. The algorithms provided by the corresponding software ActiLife (version 6.13.3) are “Troiano 2007” and the updated and modified Version “Choi 2011”. To our knowledge, the Troiano algorithm is not validated and not published in any peer-reviewed journal and is solely based on the 2003–2004 NHANES (National Health and Nutrition Examination Survey, National Cancer Institute) dataset. Choi and colleagues [9] modified this algorithm and validated it in the laboratory, however, a free-living validation is missing to this day.

The development of the algorithms was based on data collected with the Actigraph 7164 Physical Activity Monitor (Troiano) and Actigraph GT1M (Choi); however, nowadays the ActiGraph wGT3X+ is predominantly used and it is unclear if both devices use an identical data processing method. In addition, the algorithm was only validated for placement of the device on the hip but not on the wrist, which is the increasingly used placement for physical activity assessment today. To justify an even wider and more frequent use of wrist-worn accelerometers, validation of wear-time recognition under free-living rather than laboratory conditions is most important because a simulation of free-living behavior is not always possible in a laboratory setting. The primary objective of this study was, therefore, to validate the automatic wear-time recognition of wrist-worn and hip-worn ActiGraph wGT3X+ devices against self-reported non-wear time according to diaries in a free-living setting in healthy adults. The second aim was to analyze the average duration of single non-wear episodes and total duration of all non-wear episodes throughout the day. These durations are relevant to define the ideal cut-off value for the “minimum length of non-wear period” in the automatic detection of non-wear time and thereby decrease type II errors.

## Methods

This study was approved by the local ethics committee and complied with the declaration of Helsinki. Written informed consent was obtained from all study participants. Participants received two ActiGraph wGT3X+ devices. One device was worn on the wrist of the non-dominant hand and one on the opposing hip. The wearing position was entered for device initialization. Measurement rate was set at 60 Hz. Body height (cm) and body mass (kg) were measured in the laboratory and entered together with age (years) before device initialization. Participants wore the device for 3.5 consecutive days. Day 0 was not considered for data analyses as is common in the assessment of physical activity because participants are known to change their activity pattern on the initial day of data recording [10]. Day 1 and 2 were analyzed in this study. Day 3 was only recorded to ensure that participants were wearing the device through the entire night from day 2 to day 3 allowing analysis of 48 consecutive hours. Participants were advised to wear both devices continuously including during sleep times and only to remove it for showering or swimming. Additionally, participants recorded physical activity duration and intensity, sleep duration, sleep quality and non-wear time in a diary. There is no easy method to use as a gold standard for wear-time validation in a free-living setting. In this study, diary recordings were used to validate the automatic wear-time validation. Sixty-eight young, endurance-trained, male athletes were included. Although choosing athletes as the study population limits the generalizability of the results, we used this target group because it is known to be highly compliant in filling out diaries with a sufficient rigor as athletes often use training logs to keep track of their training progress. Because precise documentation of the true wear-time is indispensable to assess the accuracy of the automatic wear-time recognition reliably, this target group was favored over one that would be more representative of the general population but likely less compliant in filling out the wear-time diary. Participants were carefully instructed to record non-wear time precisely minute by minute and for each device separately. Further, participants were asked to fill out every element in the diary, so that even if they did not remove any device during the day at all, this was still recorded in the diary. This ensured that the investigators could later distinguish between missing values and wear-time compliance.

Raw data from wrist-worn and hip-worn devices were downloaded in epoch lengths of 60 s. For both wear-time validation algorithms (Troiano and Choi) default settings of ActiLife v6.13.3 were used. Both algorithms offer the option for modification such as reducing the minimum length that defines a non-wear period. However, to our knowledge, there are no publications mentioning any changes in these definitions, suggesting that researchers tend to use the default setting. There are major differences between the two algorithms. “Troiano” defines non-wear time as intervals of at least 60 consecutive minutes of zero activity counts, allowing for up to two consecutive minutes of counts between 1 and 100 counts. The algorithm “Choi” defines non-wear times as periods of consecutive 0-counts of a certain duration. This duration is defined as “minimum length of non-wear times”. The default setting by the manufacturer is 90 min. Detected non-wear times below 90 min are therefore automatically set as wear-times if this setting is not adjusted. Diaries were analyzed by summarizing non-wear time and wear-time in minutes per day for each day and device separately.

The primary outcome was the wear-time in minutes for wrist-worn and hip-worn devices calculated from the two algorithms (i.e. Troiano and Choi) compared to the respective values from the diaries. We present descriptive statistics (i.e. median and interquartile ranges) for wear times from diaries, wrist-worn and hip-worn device for each algorithm and day separately. We used paired t-test to compare the wear time from wrist-worn and hip-worn devices recorded in the diary. Additionally, we calculated Lin’s concordance correlation coefficient for each comparison [11]. All significance tests were two-sided and *P*-Values < 0.05 were considered statistically significant. We present 95% confidence intervals (95% CI) for each correlation. Further, we present Bland-Altman Plots for each comparison as well as absolute percentage errors which were calculated between wear-time according to the diary and according to the algorithm (absolute percentage errors = [(wear time ActiGraph – wear-time diary) / wear-time diary] × 100). Although the mean minutes of wear-time calculated by any algorithm may be similar to the values recorded in the diary, the correlation may still be low. This may happen when type I errors and type II errors are similar and counterbalance each other. Therefore, we further analyzed type I and type II errors for the non-wear time of the hip-worn device by the Choi algorithm in each participant. We used IBM SPSS Statistics for Windows, version 22 (IBM Corp., Armonk, N.Y., USA) and R version 3.4.2 ((R Core Team (2017): R: A language and environment for statistical computing. R Foundation for Statistical Computing, Vienna, Austria. https://www.R-project.org/)) for our analyses. No sample size calculation was performed for this explorative approach.

## Results

Participants’ mean ± standard deviation (SD) age was 25.2 ± 4.9 years, height was 180 ± 6.5 cm, body mass was 73.5 ± 6.7 kg, percent body fat mass was 11 ± 4% and body mass index was 22.7 ± 1.9 kg/m^2^. The mean ± SD values for day 1 and day 2 for vigorous physical activity were 10.9 ± 17.3 min and 10.9 ± 17.8 min, for moderate physical activity 52.3 ± 41.0 min and 52.6 ± 39.6 min, and for sedentary time 1243.8 ± 209.3 min and 1259.3 ± 203.1 min, respectively.

Table 1 shows the total wear-time in minutes recorded in the diary, wrist-worn, and hip-worn devices and the respective values calculated by the two algorithms of Troiano and Choi for each day. For wrist-worn devices, the algorithm by Troiano seems to slightly underestimate wear-time and the algorithm by Choi seems to slightly overestimate wear-time in comparison to the diary. This is visible for both days. For hip-worn devices, Troiano underestimates true wear-time to a much higher extent than it does for wrist-worn devices.

**Table 1:** Participants (*n* = 68) total wear time (min) as recorded in the diaries for wrist-worn and hip-worn devices and as calculated by the algorithms of Troiano and Choi

	Median (interquartile range) total wear time (min)	
	Day 1	Day 2
Wrist-worn		
Diary	1421 (1346, 1428)	1425 (1403, 1430)
Troiano	1402 (1296, 1440)	1406 (1334, 1440)
Choi	1440 (1386, 1440)	1440 (1440, 1440)
Hip-worn		
Diary	1421 (1334, 1428)	1425 (1400, 1430)
Troiano	1106 (990, 1210)	1116 (993, 1196)
Choi	1411 (1296, 1440)	1440 (1314, 1440)

**Fig. 1:**
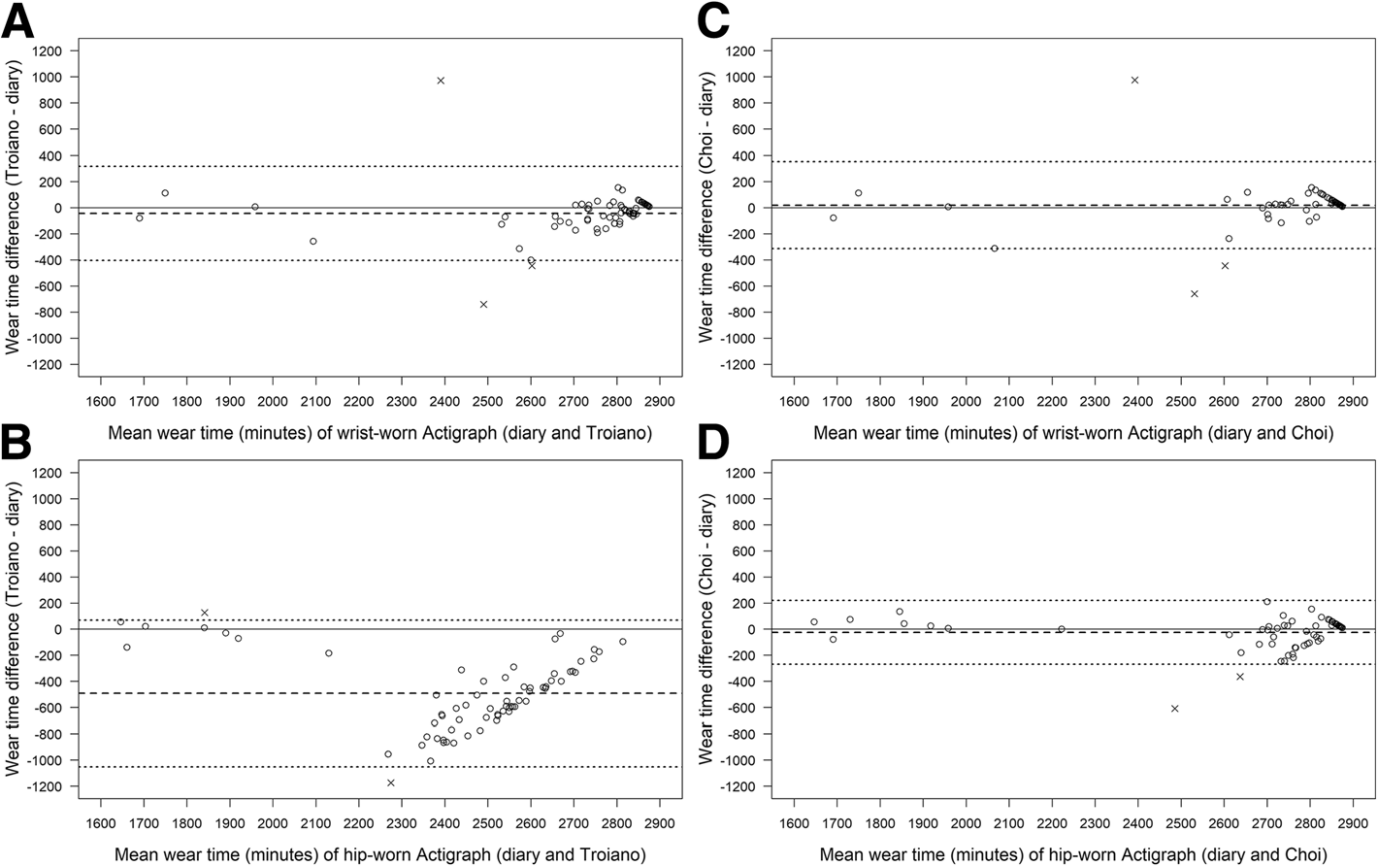
Bland–Altman plot of the total wear time (minutes) from the diaries and wrist-worn ActiGraph calculated by Troiano (**a**), wrist-worn ActiGraph calculated by Choi (**b**), hip-worn ActiGraph calculated by Troiano (**c**), hip-worn ActiGraph calculated by Choi (**d**). The dashed lines denote the bias (i.e. the mean difference) and the dotted lines denote the 95% limits of agreement

Lin’s concordance correlation coefficient for the wrist-worn wear-time was 0.73 (0.60; 0.82) when comparing the diary with Troiano and 0.78 (0.67; 0.86) when comparing the diary with Choi. For hip-worn devices the respective values were 0.23 (0.13; 0.33) for Troiano and 0.92 (0.88; 0.95) for Choi. Agreement between the methods is presented in Fig. 1 by Bland-Altman plots. Figure 1c illustrates the limited agreement between the diary and Troiano for the hip-worn Actigraph, including a negative mean difference of − 492 min and an increase of agreement with total wear-time. Mean (SD) values for absolute percentage errors for wrist-worn devices were − 1.3 ± 8.1% in Troiano and 0.9 ± 7.7% in Choi. The respective values for hip-worn devices were − 17.5 ± 10% in Troiano and − 0.8 ± 4.6% in Choi.

To illustrate the yet unaddressed problem of the definition of “minimum length of non-wear period >90 minutes” we present type I and type II errors in Table 2. Those analyses were only performed for the non-wear time as calculated by the Choi algorithm for the hip-worn device because this is currently the state of the art method to determine non-wear time.

**Table 2:** Errors for non-wear time (min) in hip-worn ActiGraph calculated by Choi-Algorithm based on non-wear time from diaries. Participants (*n* = 68)

	Error of non-wear time in min (Mean ± SD)	
	Day 1	Day 2
Type I Error		
Mean ± SD	36.8 ± 69.5	43.8 ± 76.8
Max	348	441
Type II Error		
Mean ± SD	24.9 ± 26.8	30.9 ± 33.3
Max	142	195
Total Error		
Mean ± SD	11.9 ± 70.2	12.9 ± 76.1
Min, Max	−142, 258	−195, 351

There were no significant differences in total wear-times between wrist-worn and hip-worn devices for day 1 (*p* = 0.706) or day 2 (*p* = 0.348) (see Table 1).

## Discussion

The main results of this study are that the Choi algorithm is superior to the algorithm by Troiano in hip-worn and wrist-worn devices in a free-living setting and that the automatic wear-time validation by Choi works better in hip-worn devices than in wrist-worn devices (see Fig. 1). Further, the results show that on a group level the type I, type II, and total errors are generally low in the Choi algorithm but that in some individuals the error is quite high.

The algorithm of Troiano was not valid to detect non-wear time with sufficient precision and strongly underestimated wear-time. The reason for this underestimation is that the algorithm by Troiano defines values of below 100 activity counts per minute over a period of no longer than 2 min as non-wear time although the device was worn (type I error). If an individual has an average activity count of 50 counts per minute during one activity (e.g. watching television) this would be classified as non-wear time during the entire activity by Troiano. Choi et al. (2011) reduced this value to 0 activity counts per minute. Therefore, the algorithm by Choi would detect those intensities as wear-time explaining the underestimation of wear-time by Troiano (see Table 1) and the higher precision of the Choi algorithm. The algorithm by Choi shows a high correlation for the hip-worn devices for which it was developed. Validation of free-living wear-time of the wrist placement of the ActiGraph such as that in other wrist-worn accelerometers like the GENEActiv (Activinsights, Kimbolton, United Kingdom) is missing [12]. A new wear-time validation algorithm for ActiGraph wGT3X+ devices worn on the wrist is, therefore, necessary to increase the precision of wear-time validation. Type II errors in the algorithm of Choi are mainly explained by the setting of “minimum length of non-wear period >90 minutes”. By definition, non-wear times of under 90 min cannot be detected with this setting. The intention of this definition is to reduce type I errors and to prevent exclusion of participants due to a false non-compliance. The rationale for this >90-min interval for the minimum length of non-wear time is based on a study by Choi et al. 2012 [13] in which wear-time detection was validated in a free-living setting in twenty-nine elderly subjects aged between 76 and 96 years. It is highly doubtful whether this algorithm can be applied to all target groups and correctly detect non-wear time in study samples such as ours that not only differ in activity levels but also show much different wear-time compliance and duration of non-wear epochs. In our study, seven participants on day 1 and ten participants on day 2 did not remove the ActiGraph at all. On each day, only four participants had non-wear times in episodes of more than 90 consecutive minutes as recorded in the diaries. In all of those participants, non-wear time was correctly detected by the Choi algorithm. However, the remaining participants had only non-wear episodes shorter than 90 consecutive minutes, which were subsequently not detected by the Choi algorithm. Mean and SD of type II error was 24.9 ± 26.8 min on day 1 and 30.9 ± 33.3 min on day 2 and can be classified as rather low. However, in some individuals, this error was as high as 195 min. If a person removes the device, for example, three times per day for 60 min each time, this would not be detected by the algorithm even though the overall non-wear time of three hours is quite considerable. In our highly compliant sample the average differences seem irrelevant but in a sample with a lower compliance and wear times around the cut-off for a “valid day” of 10 h, this error could be quite relevant as it could result in not detecting invalid days (i.e. < 10 h wearing time per day). Therefore, it should be discussed if the cut-off of 90 min ought to be revised and possibly reduced in studies investigating individuals with lower compliance. A reduction of this cut-off to 60 or even 30 min would admittedly result in more type I errors, however, in our participants, type I errors mainly occurred during night time. Participants that do not move a lot during nighttime have peaks of activity, but because those are often not over a period of two consecutive minutes or more, the algorithm classifies them as non-wear time. If the recording of sleep data is not the primary aim but rather the assessment of physical activity and thus wear-time during the day, a reduction of the minimum length of non-wear periods would therefore likely not lead to an increase in type I errors. A strength of this study is that we used a sample that is known to be extremely compliant in filling out diaries, as athletes often keep training logs, thus increasing the likelihood of an accurate wear-time documentation. We are aware that the choice of this target group limits the generalizability of our results. However, because no easily applicable gold standard exists, a highly compliant sample was deemed the best approach to get a first estimate of the magnitude of the wear-time recognition error.

In addition to the reduced generalizability and the lack of gold standard to determine, wear time a further limitation of this study is that the study sample is not representative with regard to its physical activity levels. The physical activity level is of relevance, because sedentary time may be misclassified as non-wear time, resulting in higher errors. Further, non-wear episodes may differ between different participants. In other populations, non-wear durations of under 90 min may appear more frequently. Therefore, our results are only able to point out the problem of defining the minimum duration of non-wear time epochs. Further validation studies focusing on the ideal cut-off to reduce type II errors without too much increasing type I errors are needed. Additionally, future validation studies need to be performed in participants with different levels of compliance because type I and type II errors affect those groups differently.

## Conclusions

In a free-living setting and participants with high compliance, the algorithm by Choi is sufficient to distinguish wear-time from non-wear time on a group level. The minimum length to define non-wear episodes has to be evaluated in further studies to define the ideal cut-off to reduce type II errors.

## Abbreviations

95% CI: 95% confidence intervals
SD: standard deviation
